# Cementless femoral neck endoprosthesis SPIRON in men in aspects of clinical status and quality of life in an average 7-year follow-up

**DOI:** 10.1186/s12891-022-05710-1

**Published:** 2022-08-03

**Authors:** Tomasz Stołtny, Bogdan Dugiełło, Michał Pyda, Jarosław Pasek, Dominika Rokicka, Marta Wróbel, Aleksander Augustyn, Daniel Spyrka, Michał Białek, Krzysztof Strojek, Bogdan Koczy

**Affiliations:** 1 District Hospital of Orthopedics and Trauma Surgery in Piekary Śląskie, Bytomska, St. 62, 41-940 Piekary Śląskie, Poland; 2 Faculty of Health Sciences, Jan Długosz University in Częstochowa, Armii Krajowej St. 13/15, 41-200 Częstochowa, Poland; 3 Department of Internal Diseases, Diabetology, and Cardiometabolic Diseases, School of Medicine with the Division of Dentistry in Zabrze, Medical University of Silesia in Katowice, Silesian Centre for Heart Diseases in Zabrze, M. Curie-Skłodowskiej 9, 41-800 Zabrze, Poland

**Keywords:** Femoral neck endoprosthesis, Hip osteoarthritis, Avascular necrosis, HHS, WOMAC, SF-12, Quality of life

## Abstract

**Background:**

We report the clinical evaluation, quality of life and pain assessment in patients who had a femoral neck SPIRON endoprosthesis.

**Methods:**

The study group consisted of 27 men in whom 35 femoral neck endoprosthesis were implanted (8 on the left side, 12 on the right side and 7 bilateral) due to idiopathic osteoarthritis of the hip (20 patients) or avascular femoral osteonecrosis (7 patients) in a mean 7-year follow-up.

**Results:**

The median pre-operative Harris Hip score (HHS) was 35.5 and post-operative 98.5 (*p* < 0.001). The median WOMAC HIP score was pre-operatively 57 and post-operatively 0 (*p* < 0.001). The median SF-12 score was pre-operatively 4 and post-operatively 33 (*p* < 0.001). The median pain assessment in VAS scale was 7 pre-operatively and 0 post-operatively (*p* < 0.001).

**Conclusions:**

The results of all examined patients have changed significantly in every category showing that SPIRON endoprosthesis improved their quality of life and statistically reduced pain ailments. Moreover we have proved that higher BMI (> 30) is associated with worse operation outcomes.

## Background

Osteoarthritis is currently the fourth leading cause of disability in women and eighth in men. Avascular osteonecrosis of the femoral head is the second cause of osteoarthritis in young patients. Among the most common causes of osteoarthritis of the hip due to avascular femoral head necrosis are: steroid therapy, alcohol abuse, chronic dialysis, femoral neck fracture, hip inflammation or immunosuppression in organ transplantation [1]. It is almost 10 times more common in the male population than earlier research results might have suggested. In young age it is the reason for giving up physical activity, absence at work and is often found in patients undergoing treatment for a “different” disease [1, 2].

Significant development of pharmacotherapy as a no-operative treatment does not bring satisfactory results. In advanced stages the only solution is arthroplasty which is intended to pursue the philosophy of ‘prosthesis before prosthesis’. In case of loosening it should successfully allow implantation of a short femoral or classic cementless endoprosthesis [2–5].

We decided to use an ultra short-stem endoprosthesis in patients with a long life expectancy due to a lower probability of loosening compared to a classic endoprosthesis. SPIRON preserves Adams arch, more bone tissue and provides a lower risk of revision surgery therefore enhancing patients quality of life [6, 7].

## Materials and methods

**Fig. 1:**
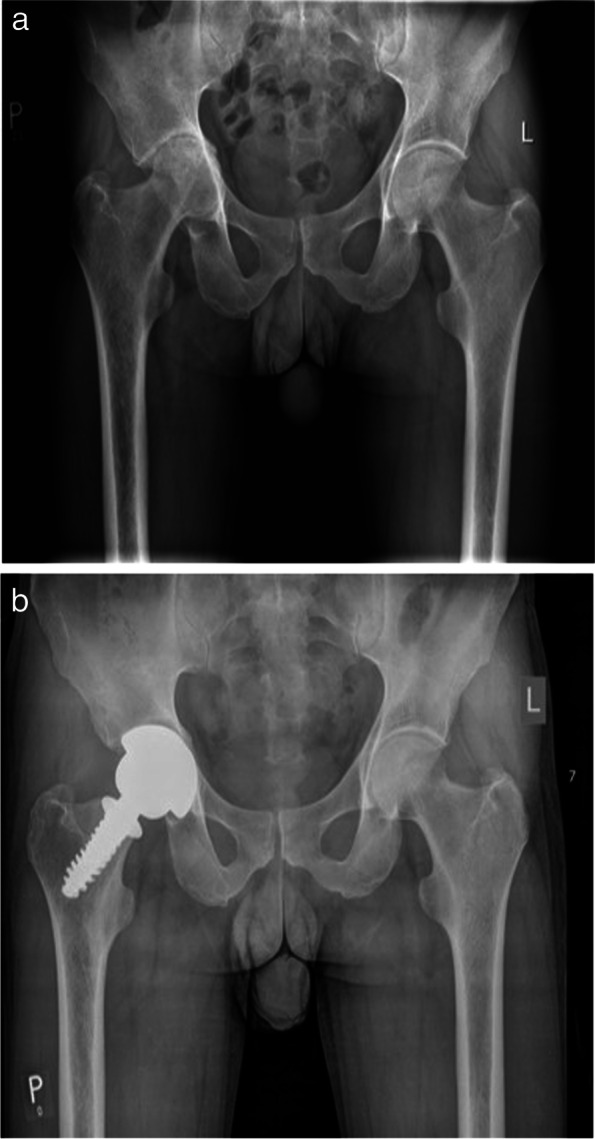
**a** Patient 42 years old diagnosed with right-sided coxarthrosis with head protrusion into the pelvis. **b** Patient 42 years old after 7 year follow-up SPIRON femoral neck endoprosthesis

**Fig. 2:**
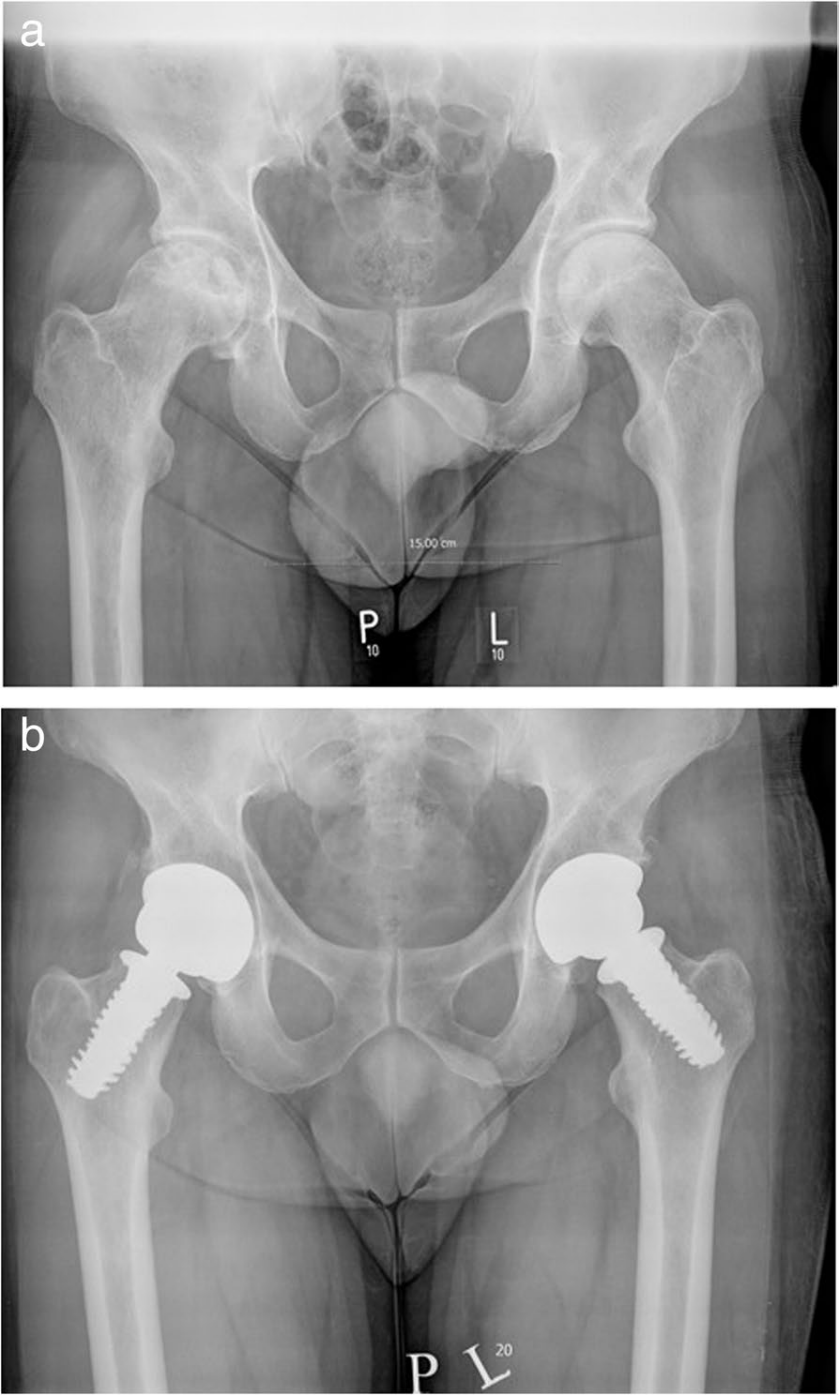
**a** Patient 54 years old patient diagnosed with bilateral degenerative changes of the hip joints secondary to AVN on the right and on the left side of femoroacetabular impingement – Femoroacetabular impingement (FAI). **b** Patient 54 years old after 7 year follow-up bilateral hip arthroplasty with the SPIRON method

**Fig. 3:**
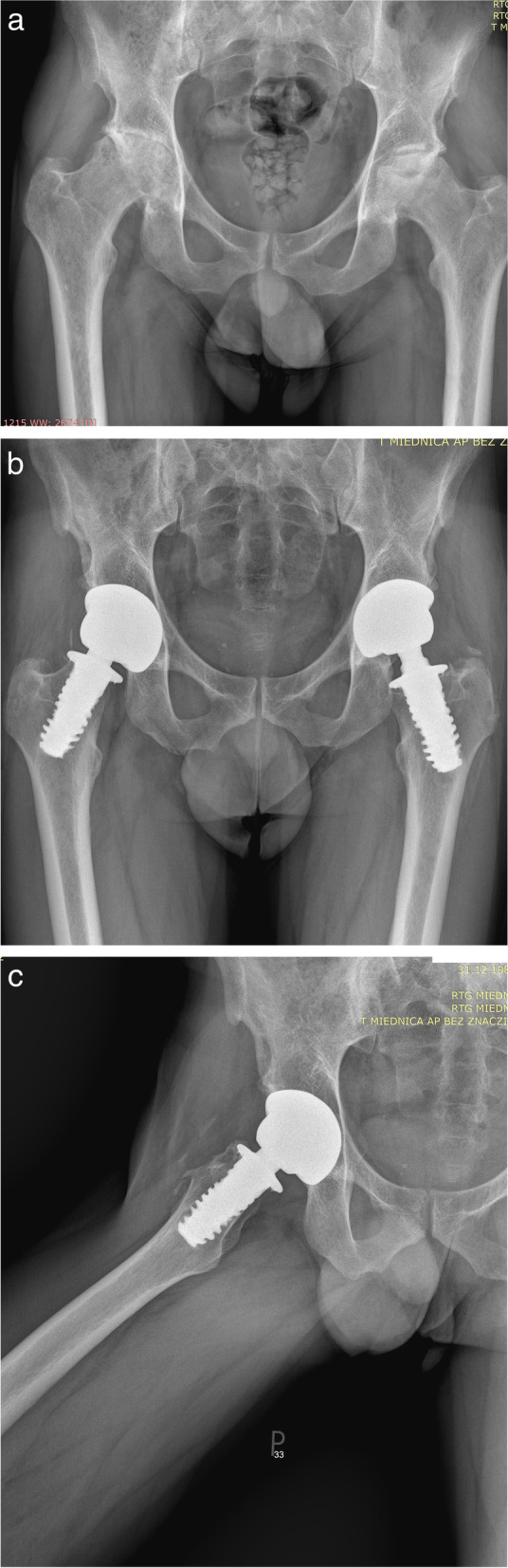
**a** Patient 34 years old diagnosed with bilateral degenerative changes of the hip joints secondary to AVN. **b** Patient 34 years old after 7 year follow-up bilateral hip arthroplasty with the SPIRON method in AP projection. **c** Patient 34 years old after 7 year follow-up bilateral hip arthroplasty with the SPIRON method in lateral projection

In the years 2012–2016 in the Department of Trauma and Orthopedics of the District Hospital of Traumatic Surgery in Piekary Śląskie, 50 femoral neck endoprosthesis in men were performed. For a follow-up examination 27 patients were reported in which 35 femoral neck endoprosthesis were implanted. The average age of patients was 46 years (from 32 to 65 years). The most common indication for surgical treatment was idiopathic osteoarthritis of the hip - 20 patients (Fig. 1a and b). The second group consisted of 7 patients with degenerative changes secondary to avascular femoral head necrosis (AVN) (Fig. 2a and b). 7 years follow-up radiological evaluation of the SPIRON stem is presented on Figs. 3a, b and c).

Inclusion criteria were: men 30–60 years old, normal geometry (CCD angle 125°-135°), normal cancellous bone density of femoral neck (assessed intra operatively), idiopathic degenerative disease, aseptic necrosis of femoral head and an informed patients’ consent. Exclusion criteria were: women (due to the ward specifics), previous femoral neck fracture using operative or no-operative methods, distorted geometry of femoral neck with multi-dimensional etiology and no patients’ consent.

SPIRON is produced by K-implant (Germany – Garbsen). It is made of bioactive titanium alloying with a calcium phosphate coating. Available sizes are 18, 20, 22, 24 mm in diameter and 50, 55, 60 and 70 mm in length [8].

In surgical treatment, Hardinge’s anterolateral approach was used with supine position. The size of the prosthesis is estimated by comparing the diameter of the femoral neck. The implantation of ceramic-on-ceramic acetabular component from titanium alloy was prepared by rasping. The femoral head was resected at cartilage-bone-border orthograde to the femoral neck axis. After reaming of the femoral neck the prosthesis should not reach the cortical bone and was screwed into the spongiosa along the femoral neck axis [8]. Threads should never reach the cortical bone. A proper Spiron implantation is well depicted on Fig. 3c. There is a high probability of a revision surgery when Spiron is not implanted along femoral axis, that is why it is a more demanding implantation technique than classic endoprosthesis.

There were no intraoperative complications and one postoperative femoral neck fracture (in this case a short stem endoprosthesis was implanted), one deep vein thrombosis and one transient femoral nerve paresis. Intraoperative medical therapy (According to the Polish Society of Orthopedics and Traumatology) included application of Biofazolin (first dose 2 g than two doses 1 g each). Post surgery management included using enoxaparin 1 day before and 30 days after the operation, neomycin in spray (it is recommended by the Polish Society of Orthopedics and Traumatology to be applied to the wound after surgery), Bioprasol, Meloxicam, Osteogenon (for 2 months to improve bone regeneration after implantation cementless endoprosthesis) and partial weight bearing for 6–8 weeks adapted to the body weight under physiotherapeutic instruction (patients begun physiotherapeutic protocols 2–3 days before the surgery). The biggest impact on physiotherapeutic protocol has the BMI and the quality of cancellous bone. Patients with high BMI or AVN are treated by our physiotherapist with special care and it may take longer for them to go from partial to full weight bearing.

Clinical condition with the HHS, WOMAC scale, quality of life on the SF-12 scale and pain VAS scale were assessed. Patients were evaluated in the average 7 year follow-up (range 4–9 years). Demographic parameters of the studied group of patients are presented in Table 1.

**Table 1:** Demographic data of the studied group of patients

*n* = 27 (35 arthroplasty performed)		
patients age	32–65 years (average 46 lat)	
BMI**	patients with AVN*	22.5 kg/cm^2^
	patients with FAI***	26.2 kg/cm^2^
Surgical treatment indications	1. idiopathic hip osteoarthritis (20 patients; bilateral - 5) 2. AVN (7 patients; bilateral - 3)	
Comorbidities	1. Rheumatoid arthritis – 2 2. Asthma bronchiale – 1 3. Ankylosing spondylitis – 1 4. Diabetes mellitus – 2 5. Hypertonia arterialis – 3 6. Sarkoidosis – 1	

AVN* (avascular femoral head necrosis); BMI** (Body Mass Index); FAI*** (femoroacetabular impingement)

The examination was conducted in accordance with the Declaration of Helsinki (1964) and its protocol was approved by the Local Bioethical Commission of the Medical University of Silesia in Katowice, Poland. All qualified patients signed written informed consent for participation in this study.

### Statistical analysis

Statistical analysis was performed using the Statistica 7.1 PL program. Values in HHS, WOMAC, VAS and SF-12 scales are unmeasurable because they describe a subjective opinion of the patient on operation outcomes. Therefore we claim that the mean average or standard deviation do not have any statistical sense using this kind of data. That is why we decided to use Wilcoxon, Sign and Trinomial tests and divided all results into first quartile (Q1), median (Me), third quartile (Q3) and also showed possible minimum (Min) and maximum (Max) values which *p* < 0.001 was considered to be statistically significant.

## Results

In the study clinical analysis on the hip joint of male patients using the following assessment scales: HHS and WOMAC was performed. Analyzing the components of the HHS classification i.e. pain and hip function improved significantly (*p* < 0.001) in both assessed parameters in every quartile and in median score (Table 2).

**Table 2:** Results of clinical evaluation of men with the HHS scale before and after SPIRON femoral neck endoprosthesis

	HHS^a^ scale					
	Pain		Function		Total	
	Before surgery	After surgery	Before surgery	After surgery	Before surgery	After surgery
Min	0	20	4	23	4	59
Q1	10	40	18,5	47	24,5	87
Me	10	44	24	55	35,5	98,5
Q3	10	44	32,75	56	43	100
Max	40	44	53	56	93	100
Wilcoxon test	*p* < 0.001		*p* < 0.001		*p* < 0.001	
n+	34		34		34	
n0	0		0		0	
n–	0		0		0	
Sign test	*p* < 0.001		*p* < 0.001		*p* < 0.001	
Trinomial test	*p* < 0.001		*p* < 0.001		*p* < 0.001	

^a^*HHS* Harris Hip Score

The stiffness, pain and activity of patients included in the WOMAC scale also reached statistical significance in the same observation period (*p* < 0.001). All patients returned to work after 2–3 months and came back to sport activities after 3–4 months. In terms of stiffness there were too many patients scoring 0 points, that is why we did not use Wilcoxon test (Table 3).

**Table 3:** Results of the clinical evaluation of men with the WOMAC scale before and after SPIRON femoral neck endoprosthesis

	WOMAC^a^ scale							
	Pain		Stiffness		Activity		Total	
	Before surgery	After surgery	Before surgery	After surgery	Before surgery	After surgery	Before surgery	After surgery
Min	2	0	0	0	3	0	5	0
Q1	10	0	0	0	36.25	0	48.25	0
Me	13	0	0	0	42.5	0	57	0
Q3	16	2	0.75	0	47	4	65.5	5.75
Max	20	12	6	3	78	36	94	50
Wilcoxon test	*p* < 0.001				*p* < 0.001		*p* < 0.001	
n+	0		0		0		0	
n0	0		29		0		0	
n–	34		5		34		34	
Sign test	*p* < 0.001		*p* = 0.031		*p* < 0.001		*p* < 0.001	
Trinomial test	*p* < 0.001		*p* = 0.022		*p* < 0.001		*p* < 0.001	

^a^*WOMAC* Western Ontario and McMaster Universities Arthritis Index

There was no significant difference (*p* > 0.001) between right and left hip arthroplasty in HHS and WOMAC scales. In addition, the quality of life and pain were assessed with the SF-12 and VAS scales, respectively. The total number of SF-12 score points improved significantly during the control examination and was statistically significantly higher (Me: 4 vs 33) compared to the pre-surgery values (p < 0.001). The assessed pain on the VAS scale decreased significantly (Me: 7 vs 0) in the control study compared to the value before surgery (p < 0.001) (Table 4).

**Table 4:** Results of quality of life assessment with the SF-12 scale and pain with the VAS scale of men after SPIRON femoral neck endoprosthesis

	VAS^a^ scale		SF-12^b^ scale	
	Before surgery	After surgery	Before surgery	After surgery
Min	3.5	0	0	0
Q1	5.625	0	0	21.25
Me	7	0	4	33
Q3	8.875	1.875	15.75	49
Max	10	6	64	64
Wilcoxon test	*p* < 0.001		*p* < 0.001	
n+	0		27	
n0	0		3	
n–	34		4	
Sign test	*p* < 0.001		*p* < 0.001	
Trinomial test	*p* < 0.001		*p* < 0.001	

^a^*VAS* Visual Analogue Scale; ^b^*SF-12* Short Form SF - 12

Among complications that occurred after surgery we found femoral neck fracture (1 patient - 3.7%), deep vein thrombosis (1 patient - 3.7%) and transient femoral nerve paresis (1 patient - 3.7%). We have also noticed that patients with higher BMI had worse operation outcomes, but there was still an improvement in their quality of life (Table 5).

**Table 5:** Table showing a positive correlation between BMI and WOMAC scale. The higher BMI, the higher WOMAC scale score and worse operation outcomes

BMI^a^	WOMAC^b^ pain	WOMAC stiffness	WOMAC activity	WOMAC total
Spearman correlation	0.391	0.291	0.379	0.386
P	0.011	0.047	0.014	0.012

^a^*BMI* Body Mass Index; ^b^*WOMAC* Western Ontario and McMaster Universities Arthritis Index

## Discussion

When choosing the right endoprosthesis for the patient in addition to medical indication factors like age, gender, a physical and professional activity of patients must be included. Considering the younger patients qualified for hip arthroplasty and their growing expectations in terms of activity and quality of life, femoral neck endoprosthesis may be increasingly used. This technique allows for a relatively high osteotomy in the femoral neck with a maximum bone preservation. However this method also has its limitations and strict indications. It can’t always be applied in situations of changed geometry of the femoral neck. Despite the lower risk of dislocation of the endoprosthesis, after this type of surgery there is a real risk of fracture of the femoral neck. Preliminary results of clinical trials are strongly encouraging as they indicate a great opportunity to undertake physical and professional activity. It can also be used in very young patients with osteoarthritis of the hip due to avascular femoral head necrosis resulting from the use of steroid drugs in the treatment of autoimmune diseases [9–11].

Tsitlakidis S. et al. after analyzing available literature (27 works) on the assessment of the clinical status of patients with osteoarthritis of the hip in whom the femoral neck endoprosthesis was used in the proximal femoral end of the hip revealed that a too short average survival of the implant (below 10 years) was the main reason for failure (revision). The authors gave two main conditions for the success of this method: lopsided femoral neck and normal bone density. Tsitlakidis and our conclusions indicate that further observation of patients after femoral stem implantation in hip arthroplasty is necessary and careful selection of patients for this type of surgery is important [12]. In our observation, after the implantation of 34 such endoprostheses, we did not observe the above-mentioned complications.

There is a lot of data indicating that a minimally invasive procedure shortens hospitalization, enables faster rehabilitation and an earlier return to full physical activity. It is also associated with less blood loss and less postoperative pain and a lower risk of infection [13–15].

In turn, in our study using anterolateral approach according to Hardinge, we obtained reduction of pain with the VAS scale of 7 points after an average follow-up of 7 years. Therefore the result obtained by us seems to be fully satisfactory. Constantly rising expectations of patients with advanced coxarthrosis, their young age and willingness to return to full activity forces engineers-constructors and orthopedic physicians to seek newer solutions in hip replacement arthroplasty like the concept of ultra-short cementless stem.

Christiansen et al. performing densitometry using the DXA method and evaluating the migration of the stem with Radiostereometric Analysis (RSA) of the PRIMOR implant in 50 patients operated within 2 years of surgery found its settling 6 weeks after surgery and clubbing occurring between 6 and 12 months after arthroplasty. They found the results to be satisfactory indicating a correlation between better bone quality and lower stem implant migration in the proximal femur in operated patients [16].

The mere use of new technologies and structures is not enough to ensure a lasting, beneficial effect of the treatment. The success of arthroplasty still depends primarily on the experience of the surgical team performing the surgery while access to modern techniques and implants allows to improve patients’ quality of life [3].

Birkhauer B. et al. in their work in a group of 38 patients over 60 years of age using the SPIRON stem neck improved their clinical condition in the early observation period (over 1 year) with the HHS scale (24 points vs. 78 points), average - 94 points. The authors performed only one revision surgery within 3 months of surgery due to early joint infection (2.63%) [6].

In turn, Lugeder A. et al. in a group of 28 patients observed a significant improvement with the HHS scale (55.4 vs 90.5) after 3 months after surgery. Only in one case there was an aseptic loosening of the stem component and a revision surgery had to be performed (3.6%). In both of the above works the authors made a clinical evaluation in the early observation period (3 months, 1 year) and obtained very good results [7]. Our assessment was carried out after an average of 7 years after surgery. We obtained a median of 98,5 points in the clinical assessment of HHS.

Keeney J. A. et al. performed retrospective pre- and postoperative demographic characteristics and functional activity profiles using classic assessment scales in two groups; under < 50 years and > 65 < 75 years in the mean follow-up period of a minimum1 year (12 months). The authors analyzed the postoperative clinical condition between the examined groups and did not find any significant differences. However, in the assessment of post-operative functional activity measured by UCLA (UCAL Loneliness Scale) classification, 37% of patients under 50 years of age and 15.5% in the group between 65 and 75 years old returned to the previously implemented activity. The above authors concluded that younger ones after hip replacement are likely to return to high functional activity. In turn, high levels of functional activity are less popular in younger patients with diagnoses other than osteoarthritis. In addition, the age of the operated patients is not a simple substitute for the level of functional activity in patients considering hip arthroplasty [17].

Cowie J. G. et al. in 239 patients assessed the impact of hip replacement on professional and sport activity showing the possibility of their resumption after a minimum of 4–6 months after the surgery. In addition, they found that higher BMI in operated patients extended this time period [18].

Oken F.O. et al. analyzed 51 professionally active patients under the age of 60 who had hip arthroplasty because of developmental dysplasia. The authors found a beneficial effect of arthroplasty on early return to work for most patients. The status of the unemployed who are ready to take up employment has also changed. In addition, carrying out endoprosthesoplasty in the above-mentioned patients increased the economic status of the region in which they worked [19].

The constantly growing number of employed patients qualified for hip replacement requires knowledge of the factors conditioning their return to work after performing the surgery and undergoing the necessary rehabilitation. The authors analyzed a group of 408 patients employed in the public sector at an average age of 54 of which 73% were women. 94% of patients employed before surgery returned to work after an average of 3 months after surgery. The identified significant factors for returning to work were: absence shorter than 30 days prior to surgery, occupied senior position and BMI less than 30. Factors such as age, gender, pre-operative health, and various health-related behaviors did not show any importance on returning to work after endoprosthesis implantation. Obese manual workers with a period of absence from work before the endoprosthesis of more than 30 days constituted a group of patients at higher risk of not returning to work after surgery [4].

Fisher N.E. et al. evaluated sport and physical activity in 117 patients after hip arthroplasty in a short observation period (2 years) showing that 87% of the patients returned to physical and sport activity after surgery [20].

Perneger T. V. et al. assessed the quality of life with the SF-12 classification in patients undergoing hip and knee replacement. The tests were carried out before surgery and 1 year after it. In the analysis, the authors pointed a significant increase in parameters with the SF-12 scale. However, it should be noted that the numerical value of the PCS (Physical Component Score) component during the follow-up evaluation after 1 year was significantly higher than the MCS (Mental Component Score) component. According to the authors carrying out a hip or knee arthroplasty a positive effect on improving the quality of life in patients is noted both, in the mental and physical sphere, although the more favorable impact of performing this procedure is more visible on a physical level [21].

The analysis carried out above using HHS, WOMAC, SF-12 and VAS scales shows that femoral neck endoprosthesis (ultra short) should be an alternative to metaphyseal (short stem) or classical cementless stem. Nowadays SPIRON is not used as a routine operative treatment because it has strict inclusion criteria and it’s implantation technique requires a longer learning curve than a classic endoprosthesis. We did not have any prior experience with Spiron because it is not a commonly used implant and there are not many clinical reports about it. We do not recommend using Spiron by young surgeons who have low experience in implanting classic endoprosthesis. The most important factors for achieving good results of SPIRON implantation are the normal femoral neck geometry and normal cancellous bone density which can be properly assessed only intraoperatively.

According to Peters RM there is a positive correlation between high BMI and worse operation outcomes what may lead to a quicker revision after primary arthroplasty. We obtained similar results, but it is worth noting that there was still a big improvement in obese patients’ (BMI > 30) quality of life [22].

Nowadays Spiron is not used as a routine operative treatment because it has strict inclusion criteria, it’s implantation technique requires a longer learning curve than a classic endoprosthesis and in our country Spiron implants got expensive over time. Moreover according to the newest trends in orthopadics short stem implants are getting more popular allowing for an easier implantation technique and a good bone preservation.

## Conclusions

1. The performance of femoral neck endoprosthesis improved the clinical condition and quality of life of the operated patients in a 7 years follow-up.
2. The use of the SPIRON femoral neck endoprosthesis reduced pain.
3. Patients with a BMI higher than normal have worse operation outcomes, but there is still an improvement in their quality of life.

## Data Availability

The datasets analyzed during the current study are not publicly available, because the collected research results were carried out on hospital patients, but are available from the corresponding author on reasonable request.

## Abbreviations

HHS: Harris Hip Score
WOMAC: Western Ontario and McMaster Universities Arthritis Index
SF-12: Short Form SF - 12
VAS: Visual Analogue Scale
AVN: Avascular Necrosis
FAI: Femoroacetabular impingement
UCLA: UCAL Loneliness Scale
BMI: Body Mass Index
PCS: Physical Component Score
MCS: Mental Component Score
